# The Endothelial Glycocalyx as a Target of Excess Soluble Fms-like Tyrosine Kinase-1

**DOI:** 10.3390/ijms24065380

**Published:** 2023-03-11

**Authors:** Annika Schulz, Carolin C. Drost, Bettina Hesse, Katrin Beul, Göran R. Boeckel, Alexander Lukasz, Hermann Pavenstädt, Marcus Brand, Giovana S. Di Marco

**Affiliations:** Department of Internal Medicine D, University Hospital Münster, 48149 Münster, Germany

**Keywords:** endothelial glycocalyx, soluble fms-like tyrosine kinase-1 (sFlt-1), heparin, endothelial dysfunction, endothelial injury, monocyte adhesion

## Abstract

Soluble fms-like tyrosine kinase-1 (sFlt-1) is a secreted protein that binds heparan sulfate expressed on the endothelial glycocalyx (eGC). In this paper we analyze how excess sFlt-1 causes conformational changes in the eGC, leading to monocyte adhesion, a key event triggering vascular dysfunction. In vitro exposure of primary human umbilical vein endothelial cells to excess sFlt-1 decreased eGC height and increased stiffness as determined by atomic force microscopy (AFM). Yet, structural loss of the eGC components was not observed, as indicated by Ulex europaeus agglutinin I and wheat germ agglutinin staining. Moreover, the conformation observed under excess sFlt-1, a collapsed eGC, is flat and stiff with unchanged coverage and sustained content. Functionally, this conformation increased the endothelial adhesiveness to THP-1 monocytes by about 35%. Heparin blocked all these effects, but the vascular endothelial growth factor did not. In vivo administration of sFlt-1 in mice also resulted in the collapse of the eGC in isolated aorta analyzed ex vivo by AFM. Our findings show that excess sFlt-1 causes the collapse of the eGC and favors leukocyte adhesion. This study provides an additional mechanism of action by which sFlt-1 may cause endothelial dysfunction and injury.

## 1. Introduction

Soluble fms-like tyrosine kinase (sFlt-1) is a non-membrane-associated vascular endothelial growth factor (VEGF) receptor. Even though sFlt-1 lacks both the transmembrane and intercellular domains, it contains the same extracellular domain and ligand-binding regions as the VEGF receptor 1 (Flt-1) [1,2,3]. In addition to VEGF (through the Ig-like domains 2–3), sFlt-1 also binds heparin (through the Ig-like domain 4) and heparan sulfate (HS) [4,5,6,7]. This property enables sFlt-1 to be locally stored in the extracellular matrix (ECM) surrounding the cell, including the endothelial glycocalyx (eGC), from where it can be displaced by heparin, a molecular mimicry of HS [8].

HS proteoglycans (HSPGs) are a major structural constituent of the eGC, a negatively charged gel-like layer coating the luminal side of the endothelium. The eGC is crucial in vascular homeostasis as a protective barrier between the blood and the endothelial membrane. Therefore, degradation or conformational changes in the eGC increases endothelial permeability, leukocyte adhesion, and dysregulated vasodilation [9,10,11,12]. In short, damage to the eGC contributes to an inflammatory response in blood vessels and causes endothelial cell dysfunction.

Excess sFlt-1 is associated with endothelial dysfunction in heart failure, sepsis, preeclampsia, chronic kidney disease, kidney transplantation, and many other diseases, including COVID-19 infection [13,14,15,16]. Interestingly, the damage to the eGC was suggested to be the leading cause of endothelial dysfunction in various cardiovascular, inflammatory, and kidney diseases [12,17,18,19]. However, our understanding of whether sFlt-1 can cause eGC dysfunction rather than being just a marker of its degradation is sparse.

Hitherto, the adverse effects of sFlt-1 have been attributed to the indiscriminate neutralization of VEGF and its antiangiogenic properties. In this study we provide evidence that excess sFlt-1 leads to conformational changes in the eGC and, consequently, facilitates the adhesion of monocytes to the endothelial monolayer, thereby suggesting a novel mechanism by which sFlt-1 induces endothelial dysfunction.

## 2. Results

### 2.1. Excess sFlt-1 Leads to Conformational Changes in the eGC In Vitro and In Vivo

**Figure 1:**
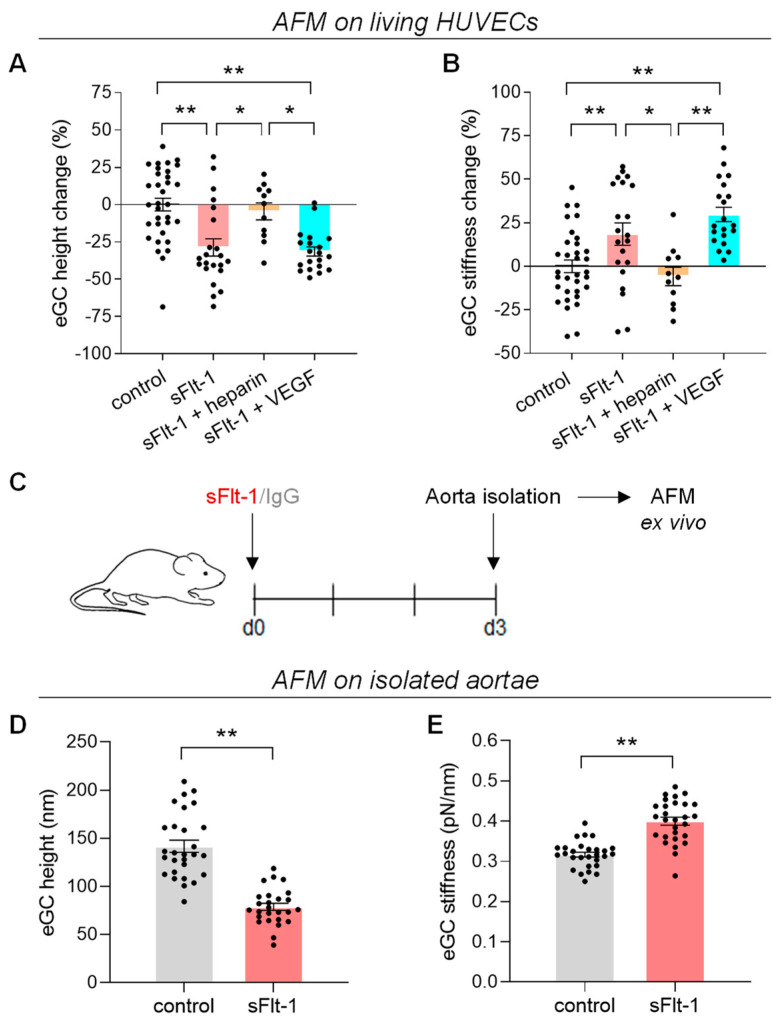
Excess sFlt-1 leads to conformational changes in the eGC in vitro and in vivo. (**A**,**B**) HUVECs were exposed to recombinant sFlt-1 (VEGFR1-Fc, 2 µg/mL) or control protein (IgG-Fc, 2 µg/mL) for 24 h in the presence or not of unfractionated heparin (10 µg/mL) after a 30 min preincubation. (**C**) Schematic representation of the experimental design for sFlt-1 delivery in vivo. Briefly, mice were exposed to continuous administration of recombinant sFlt-1 (300 ng/h; N = 3) or control protein (N = 3) for three days via osmotic minipumps implanted on day 0 (d0). Aortae were isolated on day 3 (d3), and a small patch with the endothelial surface facing upward was analyzed ex vivo (**D**,**E**). eGC height (**A**,**D**) and stiffness (**B**,**E**) were measured by atomic force microscopy (AFM). In (**A**) and (**B**), results are expressed as change relative to control. Data are given as mean ± SEM. * *p* < 0.05; ** *p* < 0.005. Nested one-way ANOVA was applied along with Tukey’s multiple comparisons test (**A**,**B**) or nested *t*-test (**D**,**E**). eGC, endothelial glycocalyx; VEGF, vascular endothelial growth factor.

The effects of sFlt-1 on the eGC in vitro and in vivo/ex vivo were analyzed using the atomic force microscopy (AFM) nanoindentation technique. Incubation of primary human umbilical vein endothelial cells (HUVECs) with recombinant human sFlt-1 (2 µg/mL) for 24 h decreased height/thickness while increasing the stiffness of the eGC; i.e., excess sFlt-1 flattens and hardens the eGC as shown in Figure 1A,B. The incubation of EA.hy926 cells, an endothelial cell line, with increasing sFlt-1 concentrations showed that these effects are dose-dependent (Figure S1). In vivo administration of recombinant sFlt-1 (300 ng/h) to mice for three days via osmotic minipump also reduced the functional height and increased the stiffness of the eGC measured ex vivo in the isolated aorta when compared to animals receiving recombinant IgG-Fc (Figure 1C,D).

Pretreatment of HUVECs with exogenous VEGF did not interfere with sFlt-1 effects on the eGC, but unfractionated heparin (10 µg/mL) completely eradicated these effects (Figure 1A,B). This result suggests that sFlt-1 effects on the eGC are mediated by the interactions between sFlt-1 and HS. By competing for the same binding sites, heparin avoids these interactions and, consequently, avoids the attachment of sFlt-1 to the eGC, as previously described [8,20]. 

**Figure 2:**
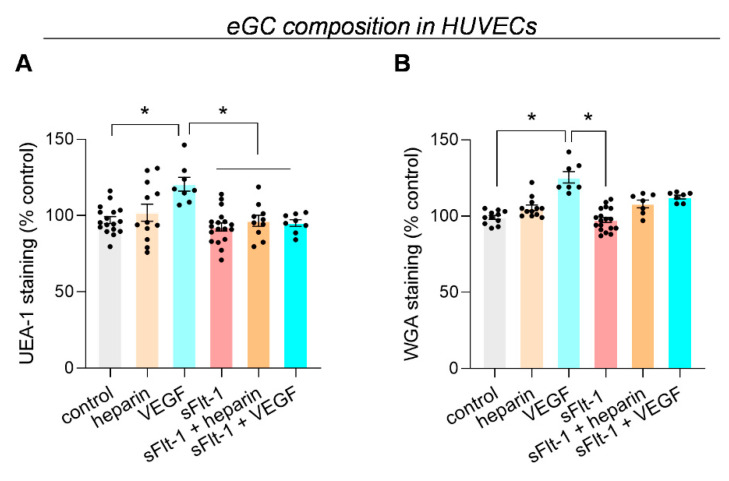
eGC composition is not altered by excess sFlt-1. HUVECs were exposed to recombinant sFlt-1 (VEGFR1-Fc, 2 µg/mL) or control protein (IgG-Fc, 2 µg/mL) for 24 h in the presence or not of unfractionated heparin (10 µg/mL) after a 30 min preincubation. Changes in the glycocalyx components were measured using the fluorescent eGC markers Ulex europaeus agglutinin-1 (UEA-1) (**A**) and wheat germ agglutinin (WGA) (**B**). Data are given as a percentage change from control and are expressed as mean ± SEM. * *p* < 0.005. Nested one-way ANOVA was applied, along with Tukey’s multiple comparisons test. eGC, endothelial glycocalyx; VEGF, vascular endothelial growth factor.

We used the fluorescent eGC markers Ulex europaeus agglutinin-1 (UEA-1) and wheat germ agglutinin (WGA) to quantify changes in the glycocalyx [21,22]. Only a slight but not significant decrease in UEA-1 fluorescence intensity was observed when HUVECs were treated with sFlt-1 (2 µg/mL) for 24 h compared to control cells. Preincubation with heparin or VEGF had no effect (Figure 2A). Additionally, similar results were observed after staining HUVECs with WGA (Figure 2B). sFlt-1 also did not change heparan sulfate distribution in EA.hy926 cells (Figure S1).

Altogether, these results indicate that rather than a structural alteration (loss of eGC components), excess sFlt-1 induces a conformational change corresponding to a collapsed eGC with decreased height and increased stiffness [9,11,12].

### 2.2. Increased Monocyte Adhesion under sFlt-1 Treatment

**Figure 3:**
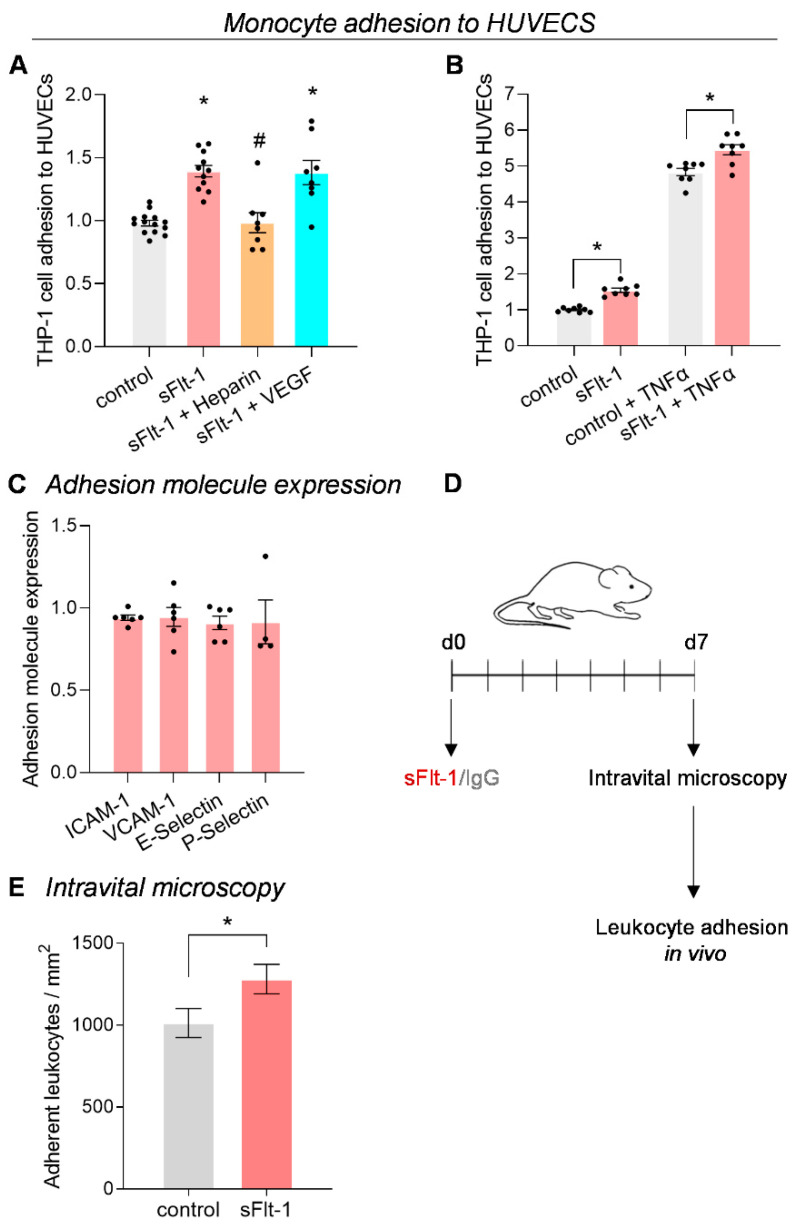
sFlt-1 increases leukocyte adhesion to the endothelium in vitro and in vivo. (**A**) HUVECs were treated with recombinant sFlt-1 (VEGFR1-Fc, 2 µg/mL) or control protein (IgG-Fc, 2 µg/mL) in the presence or not of heparin (10 µg/mL) or VEGF (50 ng/mL) for 24 h. The medium was removed, and cells were co-cultured with calcein-labeled THP-1 monocytes for 1 h. The percentage of THP-1 cells adhered to the endothelial monolayer was expressed relative to control cells. (**B**) Relative THP-1 adhesion to HUVECs treated with sFlt-1 or control protein (1 µg/mL) for 24 h and low dose tumor necrosis factor-α (TNF-α, 0.5 ng/mL) during the last 6 h. Representative results from 3 independent experiments. (**C**) Surface expression of endothelial adhesion molecules relative to control was measured by flow cytometry 4 h after exposure to sFlt-1 (2 µg/mL). ICAM-1, intercellular adhesion molecule-1; VCAM-1, vascular cell adhesion molecule-1. (**D**) Schematic representation of the experimental design for sFlt-1 delivery in vivo. Briefly, mice were exposed to continuous administration of recombinant sFlt-1 (300 ng/h) or control protein for seven days via osmotic minipumps implanted on day 0 (d0). (**E**) Intravital microscopy on postcapillary venules was performed on day 7 (d7) after 2 h of intrascrotal injection of TNF-α. Representative results from 2 independent experiments (N = 7–8 mice/treatment). Data are given as mean ± SEM. * *p* < 0.05 vs. control; ^#^ *p* < 0.05 vs. sFlt-1 + heparin. Nested one-way ANOVA was applied along with Tukey’s multiple comparisons (**A**) or Mann–Whitney test (**B**,**E**). VEGF, vascular endothelial growth factor.

A collapsed eGC characterizes a dysfunctional endothelium that favors leukocyte adhesion [9,11]. Accordingly, treatment of HUVECs with sFlt-1 (2 µg/mL) for 24 h increased the attachment rate of THP-1 monocytes by about 35% compared to control cells (Figure 3A). This effect was also observed after incubation with a lower sFlt-1 concentration (1 µg/mL) (Figure 3B). However, there were no differences in monocyte transmigration across endothelial cells treated with excess sFlt-1 (Figure S2).

Yet flow cytometric analysis revealed that the expression of adhesion molecules, including intercellular adhesion molecule-1 (ICAM-1), vascular cell adhesion molecule -1 (VCAM-1), E-Selectin, and P-Selectin, on the surface of HUVECs remained unchanged after treatment with excess sFlt-1 (Figure 3C). These findings suggest that the higher rate of monocyte adhesion observed upon sFlt-1 treatment may result from its effects on the eGC.

Accordingly, pretreatment with heparin before incubation with sFlt-1 inhibited monocyte adhesion to the endothelial monolayer, while pretreatment with VEGF did not (Figure 3A). Since sFlt-1 also interacts with α5β1 integrin in the ECM in a VEGF-independent way [23,24,25,26], we tested whether this pathway could be involved. As shown in Figure S3, function-blocking antibodies to the integrins failed to hinder the effect of sFlt-1 on the adhesion of THP-1 monocytes to HUVECs. Interestingly, we could confirm that excess sFlt-1 enhanced the impact of tumor necrosis factor-α (TNF-α) on monocyte-endothelial adhesion [27]. In vitro treatment of HUVECs with sFlt-1 (1 µg/mL) for 24 h before stimulation with a low dose of TNF-α (0.5 ng/mL) during the last 6 h further increased THP-1 adhesion in comparison to treatment with TNF-α alone (Figure 3B). 

This effect was also observed in vivo. Intravital microscopy evidenced increased adherent leukocytes to the endothelium in the TNF-α-treated cremaster muscle of mice receiving recombinant sFlt-1 (300 ng/h) compared to mice receiving control protein (Figure 3D,E).

### 2.3. Binding of sFlt-1 to the eGC and Endothelial Cell Viability

**Figure 4:**
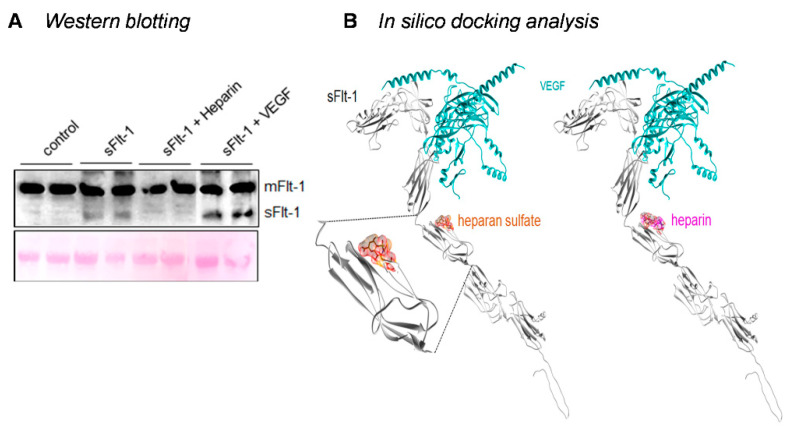
Binding of sFlt-1 to the eGC. (**A**) Representative immunoblot of HUVECs treated with sFlt-1 or control protein (2 µg/mL) in the presence or not of heparin (10 µg/mL) or exogenous VEGF (50 ng/mL) for 24 h after a 30 min preincubation. Cells were harvested with trypsin-EDTA, lysed under reducing conditions, and protein samples were blotted with an antibody that recognizes the membrane-bound receptor (mFlt-1; higher bands) and its soluble forms (sFlt-1; lower bands). By competing for the same binding site, heparin avoids the attachment of sFlt-1 to the cell membrane, while VEGF does not. Ponceau staining was used to access equal loading. (**B**) sFlt-1 interactions with VEGF (VEGF-165) through the Ig-like domain 2 and heparan sulfate and heparin through the Ig-like domain 4. Proteins are represented as gray and cyan ribbons, while ligands are orange and magenta sticks. Images were generated using the UCSF Chimera interface. eGC, endothelial glycocalyx; VEGF, vascular endothelial growth factor.

As already described above, heparin protected against sFlt-1-induced eGC damage, while preincubation with exogenous VEGF did not, suggesting that, in this case, sFlt-1 effects may be independent of VEGF signaling inhibition. sFlt-1 binds both heparin and VEGF, but only heparin seems to inhibit the interaction of sFlt-1 with the endothelial cell membrane by competing with the HS binding sites. Adherent HUVECs treated with sFlt-1 (2 µg/mL) in the presence or not of heparin or VEGF were harvested with trypsin-EDTA (without scraping or lysis agents) to avoid contamination with protein of the extracellular membrane [28], and analyzed by Western blotting. The monoclonal antibody used recognizes the full-length receptor and its truncated forms. As seen in Figure 4A, a higher molecular weight band corresponding to the endogenous, membrane-bound Flt-1 is observed in all samples. However, the lower band corresponding to the exogenous sFlt-1 is absent in heparin-treated cells and control HUVECs. Figure 4B shows the molecular interactions between sFlt-1 and its different ligands determined by in silico protein-ligand modeling. 

**Figure 5:**
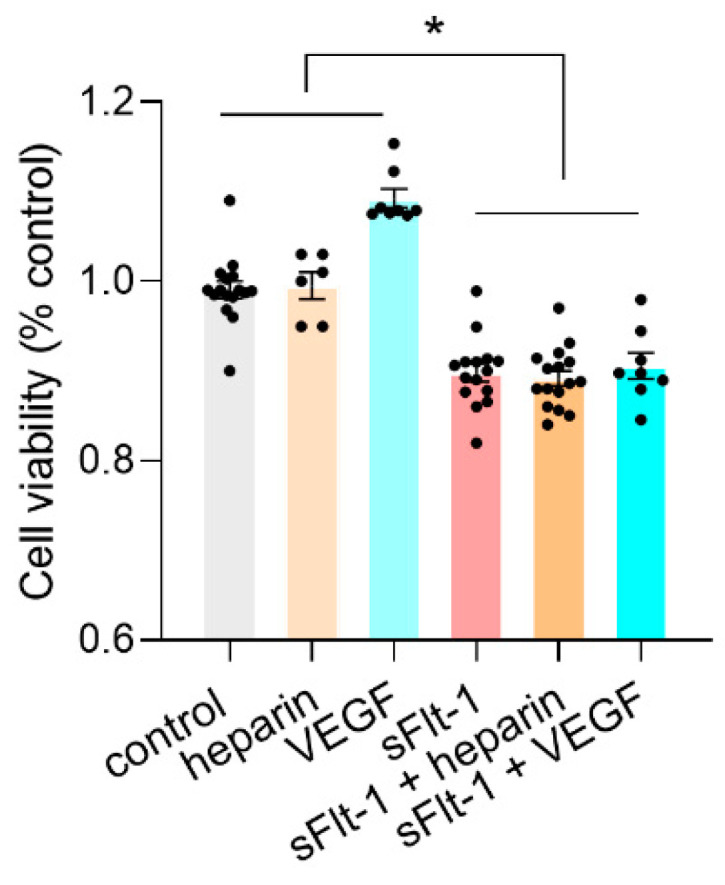
Cell viability under excess sFlt-1. HUVECs were treated with recombinant sFlt-1 (VEGFR1-Fc, 2 µg/mL) or control protein (IgG-Fc, 2 µg/mL) for 24 h in the presence or not of heparin (10 µg/mL) or VEGF (50 ng/mL) after a 30 min preincubation. Cell viability was determined using the MTT assay. Data are given as percentage change from control and expressed as mean ± SEM. Results are based on two independent experiments. * *p* < 0.05. Nested one-way ANOVA was applied along with Tukey’s multiple comparisons test. VEGF, vascular endothelial growth factor.

**Figure 6:**
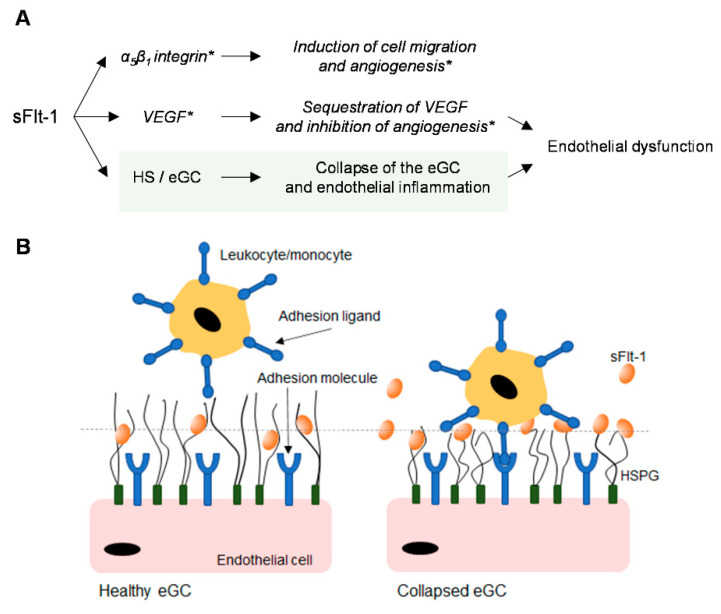
Different mechanisms of action of sFlt-1. (**A**) Depending on its interactions, sFlt-1 acts as a positive [23,25,26] or negative [1,3] regulator of angiogenesis (* based on the literature review). An additional mechanism proposed in this study (light green) is the regulation of endothelial glycocalyx (eGC) conformation through the binding of excess sFlt-1 to heparan sulfate (HS) on the cell surface; (**B**) Collapse of the eGC (decreased height and increased stiffness) favors leukocyte adhesion to the endothelium and drives endothelial inflammation and dysfunction. HSPG, heparan sulfate proteoglycans; VEGF, vascular endothelial growth factor.

Even though heparin interferes with the interaction between sFlt-1 and HS and preserves the glycocalyx from collapsing, it had no protective effects on the viability of HUVECs under excess sFlt-1 (Figure 5 and Figure S3). Figure 6 summarizes the mechanisms of action of sFlt-1 according to the interactions described in the literature and the additional mechanism involving the collapse of the eGC.

## 3. Discussion

The endothelium, spread in all tissues, is a highly active organ involved in hemostasis and inflammatory reactions. Several studies have shown that the eGC controls this vascular homeostasis, and that damage to the eGC is directly related to endothelial activation and dysfunction [12,29,30,31]. In this study, we have shown that excess sFlt-1 leads to the collapse of the eGC and consequently renders endothelial cells more adhesive to monocytes/leukocytes. This effect can be inhibited by preincubation with heparin but not with VEGF.

The eGC is attached to the endothelial cells through several membrane-bound proteoglycans and glycoproteins. Proteoglycans have a protein core to which negatively charged glycosaminoglycan (GAG) side chains are attached. Five types of GAG side chains exist, given that HS makes up 50–90%. The eGC forms a luminal network incorporating soluble molecules from plasma or endothelium. 

Even though sFlt-1 is a soluble protein, it can be stored locally by binding to HSPG on the eGC and in the abluminal ECM through its heparin-binding domain. Because of competitive binding at this domain, heparin can displace sFlt-1 [8,32]. Physiologically, local retention of sFlt-1 is essential for inhibiting VEGF excess, for example, in maintaining cornea avascularity. It also controls cell morphology and function in specialized pericytes independently of VEGF bioavailability [24,33]. Under excess sFlt-1 conditions, however, the eGC structure may become compressed, i.e., the proteoglycan structures that exist in an extended and flexible conformation collapse. In addition, a saturation of the HSPG with sFlt-1 interferes with the composition, size, and charge of the eGC, resulting in increased stiffness [34,35].

Numerous studies have shown that the eGC undergoes conformational changes when challenged with multiple biophysical and biochemical factors, including inflammatory factors, high salt (NaCl), and shear and compressive stresses [22,30,34,36]. A healthy, intact eGC is upright and soft, while a shed eGC is flat and soft. It results mainly from structural changes with degradation and loss of glycocalyx components. The conformation observed under sFlt-1 treatment, a collapsed eGC, is flat and stiff. There is a significant decrease in height and increased stiffness with unchanged coverage and sustained content [9,11,31]. Although endothelial cells display remarkable heterogeneity, [37,38] the ability to sense and respond to excess sFlt-1 with changes in the eGC seems to be preserved in endothelial cells originating from different vascular beds, e.g., HUVECs and aorta endothelial cells. 

Excess sFlt-1 production and elevated circulating levels (2- to 43-fold increase) have been observed in several diseases [39,40,41,42,43,44]. Under these conditions, circulating sFlt-1 levels increase and may reflect a decreased retention capacity due to sFlt-1 saturation [20]. Accordingly, Hagmann et al. have shown a positive correlation between the initial circulating sFlt-1 level and the degree of sFlt-1 increase after heparin administration (overall sFlt-1 load) in pregnant women, thus suggesting that in addition to higher serum levels, the ECM is also highly saturated with sFlt-1 molecules [45]. On the other hand, it is known that heparanase regulates sFlt-1 release, and correlations between circulating eGC components and sFlt-1 levels have already been described in an animal model of renal disease but not in preeclamptic women [18,46]. Therefore, the extent to which glycocalyx shedding contributes to sFlt-1 release should be further determined. 

The eGC forms an anti-inflammatory and anti-adhesive barrier at the endothelial cells. A thick eGC hinders leukocyte adhesion by covering and masking adhesion molecules on the cell surface. Since such molecules are typically smaller than the glycocalyx, reductions in eGC height make them apparent [10,21,34]. Interestingly, while eGC loss after treatment with heparanase facilitates monocyte adhesion to the endothelial cells at very high attachment rates (>80%), its collapse due to the treatment with high Na^+^ has a relatively moderate effect (about 37%) [9,10,11]. In our study, exposure of endothelial cells to excess sFlt-1 led to an increased attachment rate of monocytes by about 35%, compatible with the collapsed conformation described above. This effect was prevented by pretreatment with heparin but not with VEGF. It is of note that sFlt-1 did not affect the expression of adhesion molecules on the endothelial cell surface, nor did it induce monocyte activation (data not shown). 

Monocyte/leukocyte adhesion to endothelial cells is critical in modulating vascular inflammation and in mediating organ dysfunction and tissue injury [47]. Cell-to-cell interaction between leukocytes and endothelial cells induces signals in both cell types, contributing to cytokine production and gene induction that may aggravate inflammation [48,49,50]. In turn, endothelial inflammation may lead to eGC degradation and shedding, thus driving endothelial dysfunction. Given the likely multifactorial nature of pathological states associated with excess sFlt-1, we may emphasize the ability of sFlt-1 to render endothelial cells more sensitive to different factors such as TNF-α (as shown by us and others) [27] and angiotensin II [15,51].

By binding the ligand and blocking the membrane-bound receptors, sFlt-1 can act as a negative regulator of VEGF [1]. On the other hand, by interacting with α5β1 integrin, it functions as an ECM protein involved in cell adhesion and migration, not always in a VEGF-dependent way [23,24,25,26]. However, neither VEGF nor α5β1 integrin blockade overcame sFlt-1 effects on monocyte adhesion. Only heparin had this property. VEGF also binds heparan sulfate and heparin, yet sFlt-1 has a moderately higher affinity to its binding domain than VEGF [20]. The lower affinity of VEGF to heparan sulfate/heparin could be the reason for its inability to avoid sFlt-1 binding to the cell membrane. Computational modeling assuming that both proteins bind the ECM with the same affinity has shown a decrease in ECM-bound VEGF complexes in the presence of sFlt-1 [52].

Heparin blocked sFlt-1 effects on the eGC, including monocyte adhesion. Still, it did not protect the cells against the loss of viability. Accordingly, sFlt-1 seems to preserve its antiangiogenic properties even in the presence of heparin [32,53]. These findings agree with the idea that sFlt-1 may regulate distinct pathways (e.g., VEGF and α5β1), and that different mechanisms must be involved. The circumstances in which one will be more strongly favored than the other needs to be clarified. 

This study has some limitations. Experimental and theoretical evidence suggests that a thin deformable surface layer such as the glycocalyx (and small changes on it) may directly influence hydrodynamics and induce lift forces. In response to these forces, deformable cells migrate away from the vessel wall, thus reducing the chances of adhering [54,55]. Most of our study has been performed under static conditions and overlooked the critical role of the eGC in regulating cell/wall interactions under flow conditions. Another limitation is that the eGC and the endothelial cell cortex are mechanically and functionally connected. Hence, changes in cortical stiffness can be reflected as changes in eGC conformations [31,56]. In addition, changes in subendothelial stiffness (e.g., substrate, ECM stiffness) can directly regulate endothelial internal stiffness [57,58]. Interestingly, we have recently shown that excess sFlt-1 also stiffens the endothelial cell cortex [59]. However, treatment with heparin could not inhibit cortical stiffening, as it inhibited the stiffening of the eGC. Even though these results argue that different mechanisms may be involved, an interconnection between them cannot be excluded. In conclusion, our findings provide an additional mechanism of action by which sFlt-1 may contribute to endothelial dysfunction, namely, damage to the eGC. Furthermore, they also suggest a direct role of excess sFlt-1 in the modulation of a pro-inflammatory reaction, thus linking endothelial cell inflammation and dysfunction, both dangerous culprits in the development and progression of vascular disease.

## 4. Materials and Methods

### 4.1. Cell Culture and Treatment Protocols

Primary human umbilical vein endothelial cells (HUVEC, PromoCell, Heidelberg, Germany) were grown in Endothelial Cell Growth Medium containing fetal calf serum (FCS), endothelial cell growth supplement, epidermal growth factor, basic fibroblast growth factor, heparin, and hydrocortisone (Growth Medium SupplementPack, PromoCell, Heidelberg, Germany) as recommended by the manufacturer. In addition, EA.hy926 cells, a human umbilical vein cell line (ATCC), were grown in Dulbecco’s Modified Eagle Medium (DMEM; Biochrom, Berlin, Germany) containing 10% FCS, 2 mM L-glutamine, and 50 U/mL each of penicillin/streptomycin. Before stimulation, cells were washed with a heparin-free medium, and treatment was performed in the same medium. Cells were incubated with different sFlt-1 concentrations (recombinant human VEGFR1-Fc; R&D Systems, Minneapolis, MN, USA) for 4 or 24 h, as indicated in the Figure legends. The chosen concentrations are within the lower limit values tested extensively elsewhere [24,25,26,51]. ChromPure human IgG-Fc (Jackson ImmunoResearch, Cambridgeshire, UK) was used as the control protein. Heparin (unfractionated heparin, 10 µg/mL, Ratiopharm GmbH, Ulm, Germany), VEGF (recombinant human VEGF_165_, 50 ng/mL, R&D Systems, Minneapolis, MN, USA), the function-blocking antibodies to α5β1 (Anti-Integrin α5β1 Antibody, clone JBS5 10 µg/mL, Merck, Schuchardt, Germany) and β1-integrin (Purified Rat Anti-Human CD29, clone Mab 13, 10–20 µg/mL, BD Biosciences, San Jose, CA, USA) [60,61], were pre-incubated for 30–60 min before stimulation.

Endothelial cell viability was assessed after all treatments using the MTT assay. Briefly, 5 µL of MTT-solution (5 mg/mL) was added to endothelial cells grown in a 96-well plate (100 µL medium). After 3 h of incubation, the mixture was aspirated, and cells were solubilized with a lysing solution (100 µL/well; 100 mL SDS 20%, 34 mL *N*,*N*-Dimethylformamide, and 16 mL distilled water) overnight. Absorbance was read at 590 nm in a microplate reader (Tecan infinite microplate reader M200 Pro Tecan, Salzburg, Austria).

THP-1 monocytes were cultured in Roswell Park Memorial Institute 1640 (RPMI 1640; Biochrom, Berlin, Germany) containing 10% FCS, 2 mM L-glutamine, 1% penicillin/streptomycin, and 50 µM β-Mercaptoenthanol. All cells were kept at 37 °C in an atmosphere of 5% CO_2_.

### 4.2. eGC Analysis by Atomic Force Microscopy (AFM)

The functional height/thickness and stiffness of the eGC were determined by AFM nanoindentation technique as described previously [9,12]. Briefly, experiments were performed on living cells at 37 °C using a Multimode AFM (Veeco, Mannheim, Germany) and a feedback-controlled heating device. Endothelial cells were grown on 15 mm coverslips and treated as described above. 

Mouse aortas were isolated from mice receiving recombinant sFlt-1 or control protein as described below and freed from surrounding tissue. A small patch (approximately 1 mm^2^) of the whole aorta was removed and attached to Cell-Tak-coated (Corning, Tewksbury, MA, USA) glass, with the endothelial surface facing upward [12,30]. The aortae were kept in GM Medium (MEM containing 20% FCS, 1% MEM-vitamin, 1% MEM-NEAA, and 1% penicillin/streptomycin) until measurement. 

For the experiments, the cells and the aorta preparations were bathed at 37 °C in Hepes-buffered solution (140.0 mM NaCl, 5.0 mM KCl, 1.0 mM MgCl2, 1.0 mM CaCl2, 10.0 mM HEPES, pH 7.4) supplemented with 1% fetal calf serum (FCS). Details of the method are described elsewhere [12,30]. In brief, the glycocalyx properties of living endothelial cells were measured with a soft triangular cantilever with a mounted spherical tip (diameter 10 µm) and a spring constant of 0.01 N/m. Such a large tip can indent a larger area, thereby allowing the evaluation of the overall condition of the glycocalyx. Light microscopy was employed to ascertain that the tip position was neither at the nuclear nor at the junctional region of the endothelial cells [12]. Yet, this procedure was not possible in explanted aortas due to the lack of transparency of subendothelial layers. A laser beam registers the cantilever deflection during the indentation of the sample. The resulting curve is transformed into a force-versus-indentation curve using the cantilever’s spring constant and the optical lever sensitivity previously described and didactically illustrated by Wiesinger et al. [12]. The slope of a force-versus-indentation curve reflects the stiffness (expressed in pN/nm) which is necessary to indent the eGC for a certain distance. Details about the mathematical formulas and calculations are given elsewhere [12,62].

### 4.3. eGC Analysis by Lectin Staining 

Ulex europaeus agglutinin-1 (UEA-1) and wheat germ agglutinin (WGA) stainings were used to quantify changes in the glycocalyx. After treatment, HUVECs grown in 96-well plates were incubated with 5 µg/mL UEA-1 conjugated with fluorescein isothiocyanate (FITC) (Sigma-Aldrich, St. Louis, MO, USA) and 5 µg/mL 4′,6-diamidino-2-phenylindole (DAPI) for 20 min in Hank’s Balanced Salt Solution (HBSS). After washing with HBSS, the fluorescent signals of UEA-1-FITC and DAPI were monitored using 488/518 nm and 360/460 (Excitation/Emission), respectively, in the presence of 50 µL HBSS using a microplate reader (Tecan infinite microplate reader M200 Pro Tecan, Salzburg, Austria). Results are calculated by dividing the fluorescent signal of UEA-1-FITC by the DAPI signal (number of cells) [22].

For WGA staining, FITC-labelled WGA (Biomol GmbH, Hamburg, Germany) was added to HUVECs cultured in 96-well plates to a final concentration of 2 µg/mL. After 10 min of incubation in the growing medium, the staining solution was removed, cells were washed twice with HBSS, and the fluorescence signal was immediately monitored at 488/518 nm (excitation/emission) in the presence of 50 µL HBSS using a microplate reader (Tecan infinite microplate reader M200 Pro Tecan, Salzburg, Austria). The cells were kept on ice throughout the staining procedure to avoid endocytosis. Alternatively, cells were solubilized with Passive Lysis Buffer (PromoCell, Heidelberg, Germany) for 15 min under shaking, and fluorescence was measured as described above [9,22,30].

In addition, the endothelial monolayer was stained for heparan sulfate. EA.hy926 cells were grown in a μ-Slide 18 well (ibidi, Martinsried, Germany) fixed for 30 min at room temperature with 2% paraformaldehyde and 0.1% glutaraldehyde and washed with PBS. After blocking with 10% normal goat serum, cells were stained overnight for heparan sulfate (1:100 dilution, F58-10E4 clone; Amsbio, Abingdon, UK), before being washed and incubated with an anti-mouse Alexa Fluor 594 secondary antibody (1:500 dilution, Invitrogen, Waltham, MA, USA) and DAPI (5 µg/mL). The wells were filled with mounting medium (ibidi, Martinsried, Germany), and the cells were imaged with a Zeiss Observer Z1 Apotome microscope (Camara AxioCam Mrm and Zen software; Carl Zeiss, Jena, Germany).

### 4.4. Monocyte-Endothelial Adhesion Assay

Monocyte-endothelial cell adhesion was determined as previously described with minor modifications [63]. In brief, untreated THP-1 cells were labeled with calcein-AM (2 µM, eBioscience, San Diego, CA, USA) in phenol-free RPMI 1640 (washing medium) containing 5% FCS for 30 min at 37 °C protected from light, washed, and resuspended in phenol-free RPMI containing 2% FCS (binding medium). The cells (150,000/well) were then added to confluent endothelial monolayers grown in 96-well plates and treated as described above. Following 1 h incubation at 37 °C, non-adherent THP-1 cells were removed by washing 2–3 times with a pre-warmed medium. The fluorescent signals were assessed before (total signal) and after washing (adherent signal) by using a microplate reader (Tecan infinite microplate reader M200 Pro Tecan, Salzburg, Austria) in the presence of 100 µL binding medium. The percentage of THP-1 cells adhered to the endothelial monolayer was calculated by the formula: % adhesion = (adherent signal/total signal) × 100.

In some experiments, endothelial cells were incubated with sFlt-1 (1 µg/mL) or IgG-Fc (1 µg/mL) for 24 h, and tumor necrosis factor-α (TNF-α, 0.5 ng/mL, PeproTech, Cranbury, NJ, USA) was added for the last 6 h. Adhesion assay was performed as described above.

### 4.5. Flow Cytometric Determination of Adhesion Molecules

HUVECs were seeded in a 24-well plate and treated with sFlt-1 (1 µg/mL) for 4–5 h to analyze adhesion molecule expression. Cells were harvested with Accutase (Sigma-Aldrich, St. Louis, MO, USA), collected by centrifugation (200× *g*, 5 min), and resuspended in 100 µL FACS buffer (PBS with CaCl_2_ and MgCl_2_ containing 0.5% FCS and 0.5% NaN_3_) containing the following antibodies (1:50 dilution): biotin-conjugated anti-human intercellular adhesion molecule-1 (anti-ICAM-1, BioLegend, San Diego, CA, USA), PE-conjugated anti-human CD62E (anti-E-selectin, eBioscience, San Diego, CA, USA), PerCP/Cy5.5-conjugated anti-human CD62P (anti-P-selectin, BioLegend, San Diego, CA, USA), and APC-conjugated anti-human vascular cell adhesion molecule-1 (anti-VCAM-1, BioLegend, San Diego, CA, USA). Cells were incubated for 20 min at 4 °C, washed, and incubated with FITC-conjugated Streptavidin (1:50 dilution, BD Bioscience, CA, USA) for 20 min at 4 °C. Isotype-matched antibodies served as negative controls, and capture beads (Ultracomp eBeads, Invitrogen, Waltham, MA, USA) were used to set compensation. After washing, the cells were fixed in 1% PFA in FACS buffer and analyzed using the FACSCalibur flow cytometer (BD Bioscience, San Jose, CA, USA). Acquired data were processed with the FlowJo 10.6.2 Software (BD Bioscience, San Jose, CA, USA).

### 4.6. Western Blotting and In Silico Docking Analysis

For Western blotting analysis, adherent HUVECs were harvested with trypsin-EDTA (without scraping or lysis agents) to avoid contamination with proteins of the extracellular membrane [28]. After centrifugation, the cell pellet was lysed with Laemmli buffer under reducing conditions (20 µL/mL β-Mercaptoethanol), sonicated for 20 min in an ultrasound water bath, and boiled for 5 min at 95 °C. The samples were then subjected to a 4–20% precast polyacrylamide gel (Bio-Rad, München, Germany), and proteins were transferred to a Nitrocellulose membrane (GE Healthcare Europe GmbH, Freiburg, Germany). The membrane was probed with a rabbit monoclonal antibody against VEGF Receptor 1 (clone Y103, 1:500; #ab32152, Abcam, Cambridge, UK). Ponceau staining before antibody probing was used to access equal loading. After overnight incubation at 4 °C, the membrane was incubated with an anti-rabbit secondary antibody coupled to horseradish peroxidase (1:5000, Dako, Glostrup, Denmark) for 1 h at room temperature. For visualization, the membrane was exposed to a chemiluminescent substrate (LumiLight Plus; Roche, Mannheim, Germany) as recommended by the manufacturer. The signal was recorded with the Azure c600 Ultimate Western Imaging System (Biozym Scientific, Hess. Oldendorf, Germany).

Protein structures were predicted by RoseTTAfold [64] using the following protein accession numbers: P17948-2 (isoform 2 of vascular endothelial growth factor receptor 1; sFlt-1) and P15692-4 (VEGF-A, isoform VEGF165 of vascular endothelial growth factor A). Protein-protein and protein-ligand docking were generated using AutoDock Vina through the UCSF Chimera interface [65,66,67].

### 4.7. sFlt-1 Administration In Vivo and Intravital Microscopy

Experiments were approved by a governmental committee on animal welfare *Landesamt für Natur*, *Umwelt und Verbraucherschutz Nordrhein-Westfalen* (No. 81-02.04.2019.A208) and performed in accordance with animal protection guidelines of Germany.

Ten to twelve-week-old male C57BL/6 mice received recombinant mouse VEGFR1-Fc (300 ng/h; R&D Systems, Minneapolis, MN, USA) or control IgG-Fc (IgG2a Fc, 300 ng/h, Bio X Cell, Lebanon, NH, USA) diluted in NaCl by using osmotic minipumps (Alzet model 1007D; Durect Corporation, Bubb Road Cupertino, CA, USA) implanted subcutaneously (s.c.) on the back of each animal. This treatment raises serum sFlt-1 levels approximately 2-fold (4849 ± 1213 versus 2105 ± 518 pg/mL, VEGFR1-Fc versus control IgG-Fc; mean ± SD) [44]. Surgical interventions were conducted under inhalation anesthesia with a 1.5–2.5% isoflurane/oxygen mixture (Abbott GmbH & Co. KG, Wiesbaden, Germany), and buprenorphine was administered (0.05 mg/kg, s.c.) to control wound pain. The animals were maintained in a temperature-controlled chamber at 24 °C with a 12:12 h light–dark cycle on standard commercial chow and tap water ad libitum. After three days, the mice were sacrificed, and the aortae were extracted and stored in DMEM (Biochrom, Berlin, Germany) until preparation for AFM analysis, as described above.

Leukocyte adhesion to the endothelium in vivo was analyzed by intravital microscopy on postcapillary venules (diameter between 20 and 40 µm) of the cremaster muscle after seven days of sFlt-1 administration. In brief, the cremaster muscle of the anesthetized mice (i.p. injection of 125 mg/kg ketamine hydrochloride and 12.5 mg/kg xylazine) was prepared for intravital imaging as previously described [68]. Two hours before cremaster exteriorization, the mice received an intrascrotal injection of 500 ng TNF-α to induce local inflammation. Intravital microscopy was carried out on an upright microscope with a 40 × 0.75 NA saline immersion objective. Leukocyte rolling velocity and leukocyte arrest were determined by transillumination intravital microscopy, whereas leukocyte extravasation was investigated by near-infrared reflected-light oblique transillumination microscopy. Recorded images were analyzed using ImageJ (version 1.43a, National Institute of Health, https://imagej.nih.gov/ij/, accessed on 12 February 2023) and Zen software (Carl Zeiss, Jena, Germany). 

### 4.8. Statistical Analysis

Data are presented as mean ± SEM. Analyses were performed using GraphPad Prism version 9.4.1 for Windows (GraphPad Prism Software Inc., San Diego, CA, USA). The statistical tests used are given in individual Figure legends. All analyses were considered exploratory. Accordingly, *p*-values are given as descriptive measures, and the two-sided *p* < 0.05 was accepted as statistically significant.

## Data Availability

All data generated or analyzed during this study are included in the manuscript and supporting source files (Supplementary Materials). Further inquiries can be directed to the corresponding author.

## Supplementary Materials

The following supporting information can be downloaded at: https://www.mdpi.com/article/10.3390/ijms24065380/s1.

## References

[1] Kendall R.L., Thomas K.A. (1993). Inhibition of Vascular Endothelial Cell Growth Factor Activity by an Endogenously Encoded Soluble Receptor. Proc. Natl. Acad. Sci. USA.

[2] Kendall R.L., Wang G., Thomas K.A. (1996). Identification of a Natural Soluble Form of the Vascular Endothelial Growth Factor Receptor, FLT-1, and Its Heterodimerization with KDR. Biochem. Biophys. Res. Commun..

[3] Shibuya M. (2001). Structure and Dual Function of Vascular Endothelial Growth Factor Receptor-1 (Flt-1). Int. J. Biochem. Cell Biol..

[4] Wiesmann C., Fuh G., Christinger H.W., Eigenbrot C., Wells J.A., de Vos A.M. (1997). Crystal Structure at 1.7 Å Resolution of VEGF in Complex with Domain 2 of the Flt-1 Receptor. Cell.

[5] Ma L., Wang X., Zhang Z., Zhou X., Chen A., Yao L. (2001). Identification of the Ligand-Binding Domain of Human Vascular-Endothelial-Growth-Factor Receptor Flt-1. Biotechnol. Appl. Biochem..

[6] Markovic-Mueller S., Stuttfeld E., Asthana M., Weinert T., Bliven S., Goldie K.N., Kisko K., Capitani G., Ballmer-Hofer K. (2017). Structure of the Full-Length VEGFR-1 Extracellular Domain in Complex with VEGF-A. Structure.

[7] Park M., Lee S.T. (1999). The Fourth Immunoglobulin-like Loop in the Extracellular Domain of FLT-1, a VEGF Receptor, Includes a Major Heparin-Binding Site. Biochem. Biophys. Res. Commun..

[8] Moore K.H., Chapman H., George E.M. (2020). Unfractionated Heparin Displaces SFlt-1 from the Placental Extracellular Matrix. Biol. Sex Differ..

[9] Oberleithner H., Peters W., Kusche-Vihrog K., Korte S., Schillers H., Kliche K., Oberleithner K. (2011). Salt Overload Damages the Glycocalyx Sodium Barrier of Vascular Endothelium. Pflug. Arch..

[10] Schierke F., Wyrwoll M.J., Wisdorf M., Niedzielski L., Maase M., Ruck T., Meuth S.G., Kusche-Vihrog K. (2017). Nanomechanics of the Endothelial Glycocalyx Contribute to Na+—Induced Vascular Inflammation. Sci. Rep..

[11] Fels J., Kusche-Vihrog K. (2019). Endothelial Nanomechanics in the Context of Endothelial (Dys)Function and Inflammation. Antioxid. Redox Signal..

[12] Wiesinger A., Peters W., Chappell D., Kentrup D., Reuter S., Pavenstädt H., Oberleithner H., Kümpers P. (2013). Nanomechanics of the Endothelial Glycocalyx in Experimental Sepsis. PLoS ONE.

[13] Wewers T.M., Schulz A., Nolte I., Pavenstädt H., Brand M., di Marco G.S. (2021). Circulating Soluble Fms-like Tyrosine Kinase in Renal Diseases Other than Preeclampsia. J. Am. Soc. Nephrol..

[14] Greco M., Palumbo C., Sicuro F., Lobreglio G. (2018). Soluble Fms-Like Tyrosine Kinase-1 Is A Marker of Endothelial Dysfunction During Sepsis. J. Clin. Med. Res..

[15] Giardini V., Carrer A., Casati M., Contro E., Vergani P., Gambacorti-Passerini C. (2020). Increased SFLT-1/PlGF Ratio in COVID-19: A Novel Link to Angiotensin II-mediated Endothelial Dysfunction. Am. J. Hematol..

[16] Hernandez-Pacheco J.A., Torres-Torres J., Martinez-Portilla R.J., Solis-Paredes J.M., Estrada-Gutierrez G., Mateu-Rogell P., Nares-Torices M.A., Lopez-Marenco M.E., Escobedo-Segura K.R., Posadas-Nava A. (2022). SFlt-1 Is an Independent Predictor of Adverse Maternal Outcomes in Women With SARS-CoV-2 Infection and Hypertensive Disorders of Pregnancy. Front. Med..

[17] Ziganshina M.M., Yarotskaya E.L., Bovin N.V., Sukhikh G.T. (2018). Endothelial Dysfunction as a Consequence of Endothelial Glycocalyx Damage: A Role in the Pathogenesis of Preeclampsia. Endothelial Dysfunction—Old Concepts and New Challenges.

[18] Padberg J.S., Wiesinger A., di Marco G.S., Reuter S., Grabner A., Kentrup D., Lukasz A., Oberleithner H., Pavenstädt H., Brand M. (2014). Damage of the Endothelial Glycocalyx in Chronic Kidney Disease. Atherosclerosis.

[19] Weissgerber T.L., Garcia-Valencia O., Milic N.M., Codsi E., Cubro H., Nath M.C., White W.M., Nath K.A., Garovic V.D. (2019). Early Onset Preeclampsia Is Associated with Glycocalyx Degradation and Reduced Microvascular Perfusion. J. Am. Heart Assoc..

[20] Sela S., Natanson-Yaron S., Zcharia E., Vlodavsky I., Yagel S., Keshet E. (2011). Local Retention versus Systemic Release of Soluble VEGF Receptor-1 Are Mediated by Heparin-Binding and Regulated by Heparanase. Circ. Res..

[21] Dragovich M.A., Genemaras K., Dailey H.L., Jedlicka S., Frank Zhang X. (2017). Dual Regulation of L-Selectin-Mediated Leukocyte Adhesion by Endothelial Surface Glycocalyx. Cell. Mol. Bioeng..

[22] Pollmann S., Scharnetzki D., Manikowski D., Lenders M., Brand E. (2021). Endothelial Dysfunction in Fabry Disease Is Related to Glycocalyx Degradation. Front. Immunol..

[23] Colotti G., Failla C.M., Lacal P.M., Ungarelli M., Ruffini F., di Micco P., Orecchia A., Morea V. (2022). Neuropilin-1 Is Required for Endothelial Cell Adhesion to Soluble Vascular Endothelial Growth Factor Receptor 1. FEBS J..

[24] Jin J., Sison K., Li C., Tian R., Wnuk M., Sung H.K., Jeansson M., Zhang C., Tucholska M., Jones N. (2012). Soluble FLT1 Binds Lipid Microdomains in Podocytes to Control Cell Morphology and Glomerular Barrier Function. Cell.

[25] Orecchia A., Lacal P.M., Schietroma C., Morea V., Zambruno G., Failla C.M. (2003). Vascular Endothelial Growth Factor Receptor-1 Is Deposited in the Extracellular Matrix by Endothelial Cells and Is a Ligand for the A5β1 Integrin. J. Cell Sci..

[26] Soro S., Orecchia A., Morbidelli L., Lacal P.M., Morea V., Ballmer-Hofer K., Ruffini F., Ziche M., D’Atri S., Zambruno G. (2008). A Proangiogenic Peptide Derived from Vascular Endothelial Growth Factor Receptor-1 Acts through Alpha5beta1 Integrin. Blood.

[27] Cindrova-Davies T., Sanders D.A., Burton G.J., Charnock-Jones D.S. (2011). Soluble FLT1 Sensitizes Endothelial Cells to Inflammatory Cytokines by Antagonizing VEGF Receptor-Mediated Signalling. Cardiovasc. Res..

[28] Naba A., Clauser K.R., Hynes R.O. (2015). Enrichment of Extracellular Matrix Proteins from Tissues and Digestion into Peptides for Mass Spectrometry Analysis. J. Vis. Exp..

[29] Vlahu C.A., Lemkes B.A., Struijk D.G., Koopman M.G., Krediet R.T., Vink H. (2012). Damage of the Endothelial Glycocalyx in Dialysis Patients. J. Am. Soc. Nephrol..

[30] Hesse B., Rovas A., Buscher K., Kusche-Vihrog K., Brand M., di Marco G.S., Kielstein J.T., Pavenstädt H., Linke W.A., Nofer J.R. (2020). Symmetric Dimethylarginine in Dysfunctional High-Density Lipoprotein Mediates Endothelial Glycocalyx Breakdown in Chronic Kidney Disease. Kidney Int..

[31] Cosgun Z.C., Fels B., Kusche-Vihrog K. (2020). Nanomechanics of the Endothelial Glycocalyx: From Structure to Function. Am. J. Pathol..

[32] Searle J., Mockel M., Gwosc S., Datwyler S.A., Qadri F., Albert G.I., Holert F., Isbruch A., Klug L., Muller D.N. (2011). Heparin Strongly Induces Soluble Fms-like Tyrosine Kinase 1 Release in Vivo and in Vitro-Brief Report. Arterioscler. Thromb. Vasc. Biol..

[33] Ambati B.K., Nozaki M., Singh N., Takeda A., Jani P.D., Suthar T., Albuquerque R.J.C., Richter E., Sakurai E., Newcomb M.T. (2006). Corneal Avascularity Is Due to Soluble VEGF Receptor-1. Nature.

[34] Gandhi J.G., Koch D.L., Paszek M.J. (2019). Equilibrium Modeling of the Mechanics and Structure of the Cancer Glycocalyx. Biophys. J..

[35] Siren E.M.J., Chapanian R., Constantinescu I., Brooks D.E., Kizhakkedathu J.N. (2018). Oncotically Driven Control over Glycocalyx Dimension for Cell Surface Engineering and Protein Binding in the Longitudinal Direction. Sci. Rep..

[36] Delgadillo L.F., Lomakina E.B., Kuebel J., Waugh R.E. (2021). Changes in Endothelial Glycocalyx Layer Protective Ability after Inflammatory Stimulus. Am. J. Physiol. Cell Physiol..

[37] Lau S., Gossen M., Lendlein A., Jung F. (2021). Venous and Arterial Endothelial Cells from Human Umbilical Cords: Potential Cell Sources for Cardiovascular Research. Int. J. Mol. Sci..

[38] McCarron J.G., Lee M.D., Wilson C. (2017). The Endothelium Solves Problems That Endothelial Cells Do Not Know Exist. Trends Pharmacol. Sci..

[39] di Marco G.S., Reuter S., Hillebrand U., Amler S., König M., Larger E., Oberleithner H., Brand E., Pavenstädt H., Brand M. (2009). The Soluble VEGF Receptor SFlt1 Contributes to Endothelial Dysfunction in CKD. J. Am. Soc. Nephrol..

[40] Ky B., French B., Ruparel K., Sweitzer N.K., Fang J.C., Levy W.C., Sawyer D.B., Cappola T.P. (2011). The Vascular Marker Soluble Fms-Like Tyrosine Kinase 1 Is Associated with Disease Severity and Adverse Outcomes in Chronic Heart Failure. J. Am. Coll. Cardiol..

[41] Di Marco G.S., Kentrup D., Reuter S., Mayer A.B., Golle L., Tiemann K., Fobker M., Engelbertz C., Breithardt G., Brand E. (2015). Soluble Flt-1 Links Microvascular Disease with Heart Failure in CKD. Basic Res. Cardiol..

[42] Maynard S.E., Min J.Y., Merchan J., Lim K.H., Li J., Mondal S., Libermann T.A., Morgan J.P., Sellke F.W., Stillman I.E. (2003). Excess Placental Soluble Fms-like Tyrosine Kinase 1 (SFlt1) May Contribute to Endothelial Dysfunction Hypertension, and Proteinuria in Preeclampsia. J. Clin. Investig..

[43] Wikström A.K., Larsson A., Eriksson U.J., Nash P., Nordén-Lindeberg S., Olovsson M. (2007). Placental Growth Factor and Soluble FMS-like Tyrosine Kinase-1 in Early-Onset and Late-Onset Preeclampsia. Obstet. Gynecol..

[44] Wewers T.M., Mayer A.B., Pfleiderer A., Beul K., Schmidt R., Heitplatz B., van Marck V., Nolte I., Pavenstädt H., Reuter S. (2019). Increased Soluble Fms-like Tyrosine Kinase 1 after Ischemia Reperfusion Contributes to Adverse Clinical Outcomes Following Kidney Transplantation. Kidney Int..

[45] Hagmann H., Bossung V., Ali Belaidi A., Fridman A., Karumanchi S.A., Thadhani R., Schermer B., Mallmann P., Schwarz G., Benzing T. (2014). Low-Molecular Weight Heparin Increases Circulating SFlt-1 Levels and Enhances Urinary Elimination. PLoS ONE.

[46] Kornacki J., Wirstlein P., Wender-Ozegowska E. (2021). Serum Levels of Soluble FMS-like Tyrosine Kinase 1 and Endothelial Glycocalyx Components in Early- and Late-Onset Preeclampsia. J. Matern. Fetal Neonatal Med..

[47] Hilgendorf I., Swirski F.K., Robbins C.S. (2015). Monocyte Fate in Atherosclerosis. Arterioscler. Thromb. Vasc. Biol..

[48] Huang A.J., Manning J.E., Bandak T.M., Ratau M.C., Hanser K.R., Silverstein S.C. (1993). Endothelial Cell Cytosolic Free Calcium Regulates Neutrophil Migration across Monolayers of Endothelial Cells. J. Cell Biol..

[49] Eiermann D.F., Johnson C.E., Haskill J.S. (1989). Human Monocyte Inflammatory Mediator Gene Expression Is Selecively Regulated by Adherence Substrates. J. Immunol..

[50] Sun H.J., Wu Z.Y., Nie X.W., Bian J.S. (2020). Role of Endothelial Dysfunction in Cardiovascular Diseases: The Link between Inflammation and Hydrogen Sulfide. Front. Pharmacol..

[51] Burke S.D., Zsengellér Z.K., Khankin E.V., Lo A.S., Rajakumar A., DuPont J.J., McCurley A., Moss M.E., Zhang D., Clark C.D. (2016). Soluble FMS-like Tyrosine Kinase 1 Promotes Angiotensin II Sensitivity in Preeclampsia. J. Clin. Investig..

[52] Hashambhoy Y.L., Chappell J.C., Peirce S.M., Bautch V.L., mac Gabhann F. (2011). Computational Modeling of Interacting VEGF and Soluble VEGF Receptor Concentration Gradients. Front. Physiol..

[53] Rosenberg V.A., Buhimschi I.A., Lockwood C.J., Paidas M.J., Dulay A.T., Ramma W., Abdel-Razeq S.S., Zhao G., Ahmad S., Ahmed A. (2011). Heparin Elevates Circulating Soluble Fms-like Tyrosine Kinase-1 Immunoreactivity in Pregnant Women Receiving Anticoagulation Therapy. Circulation.

[54] Geislinger T.M., Franke T. (2014). Hydrodynamic Lift of Vesicles and Red Blood Cells in Flow—From Fåhræus & Lindqvist to Microfluidic Cell Sorting. Adv. Colloid Interface Sci..

[55] Davies H.S., Débarre D., El Amri N., Verdier C., Richter R.P., Bureau L. (2018). Elastohydrodynamic Lift at a Soft Wall. Phys. Rev. Lett..

[56] Peters W., Kusche-Vihrog K., Oberleithner H., Schillers H. (2015). Cystic Fibrosis Transmembrane Conductance Regulator Is Involved in Polyphenol-Induced Swelling of the Endothelial Glycocalyx. Nanomedicine.

[57] Bastounis E.E., Yeh Y.T., Theriot J.A. (2019). Subendothelial Stiffness Alters Endothelial Cell Traction Force Generation While Exerting a Minimal Effect on the Transcriptome. Sci. Rep..

[58] Gordon E., Schimmel L., Frye M. (2020). The Importance of Mechanical Forces for in Vitro Endothelial Cell Biology. Front. Physiol..

[59] Schulz A., Drost C.C., Hesse B., Beul K., Brand M., Di Marco G.S. (2022). The Soluble Fms-like Tyrosine Kinase-1 Contributes to Structural and Functional Changes in Endothelial Cells in Chronic Kidney Disease. Int. J. Mol. Sci..

[60] Calzada M.J., Annis D.S., Zeng B., Marcinkiewicz C., Banas B., Lawler J., Mosher D.F., Roberts D.D. (2004). Identification of Novel Β1 Integrin Binding Sites in the Type 1 and Type 2 Repeats of Thrombospondin-1. J. Biol. Chem..

[61] Humphries J.D., Schofield N.R., Mostafavi-Pour Z., Green L.J., Garratt A.N., Mould A.P., Humphries M.J. (2005). Dual Functionality of the Anti-Β1 Integrin Antibody, 12G10, Exemplifies Agonistic Signalling from the Ligand Binding Pocket of Integrin Adhesion Receptors. J. Biol. Chem..

[62] Fels J., Jeggle P., Kusche-Vihrog K., Oberleithner H. (2012). Cortical Actin Nanodynamics Determines Nitric Oxide Release in Vascular Endothelium. PLoS ONE.

[63] Golle L., Gerth H.U., Beul K., Heitplatz B., Barth P., Fobker M., Pavenstädt H., di Marco G.S., Brand M. (2017). Bone Marrow-Derived Cells and Their Conditioned Medium Induce Microvascular Repair in Uremic Rats by Stimulation of Endogenous Repair Mechanisms. Sci. Rep..

[64] Baek M., DiMaio F., Anishchenko I., Dauparas J., Ovchinnikov S., Lee G.R., Wang J., Cong Q., Kinch L.N., Dustin Schaeffer R. (2021). Accurate Prediction of Protein Structures and Interactions Using a Three-Track Neural Network. Science.

[65] Eberhardt J., Santos-Martins D., Tillack A.F., Forli S. (2021). AutoDock Vina 1.2.0: New Docking Methods, Expanded Force Field, and Python Bindings. J. Chem. Inf. Model..

[66] Trott O., Olson A.J. (2010). AutoDock Vina: Improving the Speed and Accuracy of Docking with a New Scoring Function, Efficient Optimization, and Multithreading. J. Comput. Chem..

[67] Pettersen E.F., Goddard T.D., Huang C.C., Couch G.S., Greenblatt D.M., Meng E.C., Ferrin T.E. (2004). UCSF Chimera—A Visualization System for Exploratory Research and Analysis. J. Comput. Chem..

[68] Stadtmann A., Block H., Volmering S., Abram C., Sohlbach C., Boras M., Lowell C.A., Zarbock A. (2015). Cross-Talk between Shp1 and PIPKIγ Controls Leukocyte Recruitment. J. Immunol..

