# Supplementary material for: The Endothelial Glycocalyx as a Target of Excess Soluble Fms-like Tyrosine Kinase-1
Source: Int J Mol Sci. 2023 Mar 11;24(6):5380. doi: 10.3390/ijms24065380 (PMC10049398; doi:10.3390/ijms24065380)
Supplement: Supplementary file 1 [file ijms-24-05380-s001.zip › ijms-2248567-supplementary.pdf]

Supplementary Material

The endothelial glycocalyx as a target of excess soluble fms-like tyrosine kinase-1

Annika Schulz [1], Carolin C. Drost [1] Bettina Hesse [1], Katrin Beul [1], Göran R. Boeckel [1], Alexander Lukasz [1], Hermann Pavenstädt [1], Marcus Brand [1] and Giovana S. Di Marco [1,*]

Department of Internal Medicine D, University Hospital Münster, 48149 Münster, Germany;
*Correspondence: giodimarco@gmail.com; Tel.: +49 251 8356911

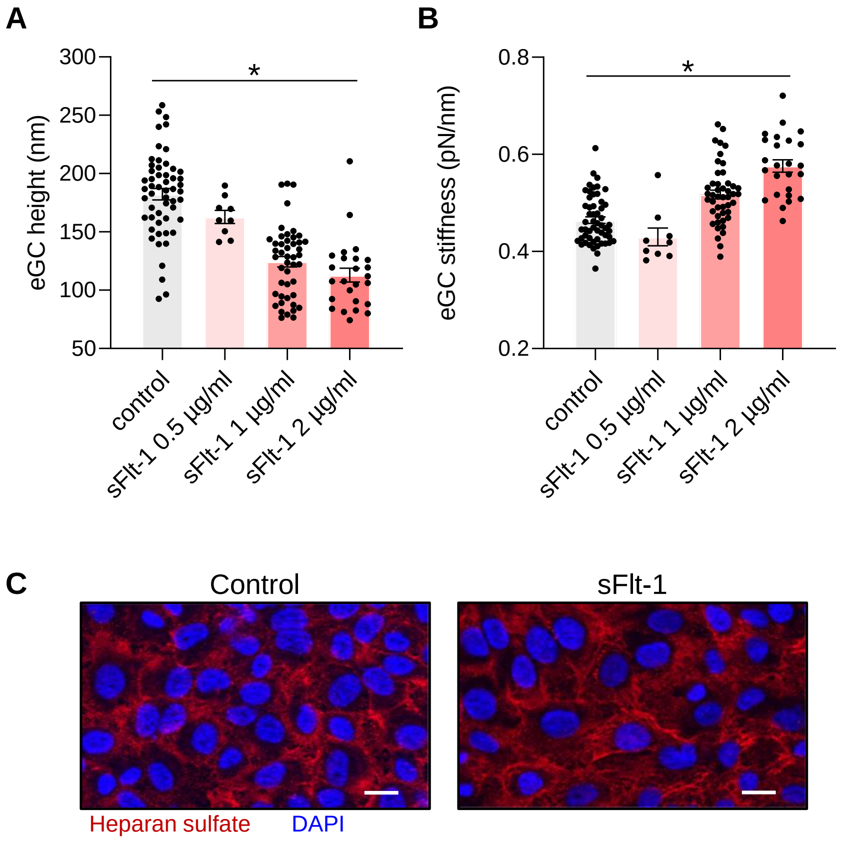

Figure S1. sFlt-1 leads to conformational changes of the eGC in a dose-dependent manner. EA.hy926 cells were cultured with increasing sFlt-1 (VEGFR1-Fc) concentrations for 24 h. Control cells were incubated with control protein (IgG-Fc, 1 µg/ml). eGC height (**A**) and stiffness (**B**) were measured by atomic force microscopy (AFM). (**C**) Representative immunofluorescence images of EA.hy926 cells treated with sFlt-1 (2 µg/ml; left panel) or control protein (2 µg/ml; right panel) and stained for anti-heparan sulfate. DAPI indicates nuclear staining. Data are given as mean ± SEM. * p <0.001 for linear trend determined by one-way ANOVA. eGC, endothelial glycocalyx

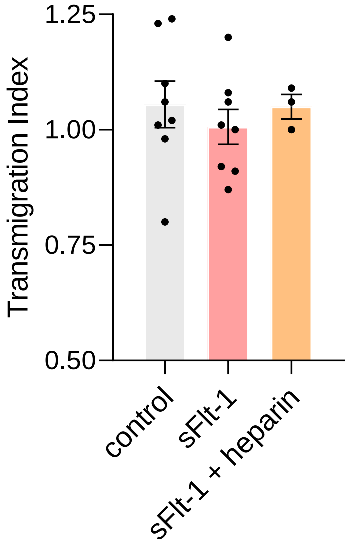

Figure S2. Monocyte transmigration across endothelial cells exposed to excess sFlt-1. HUVECs were cultured in transwell inserts (5 µm pore; 25,000 cells/insert) and treated with recombinant sFlt-1 (VEGFR1-Fc, 2 µg/ml) or control protein (IgG-Fc, 2 µg/ml) for 24h in the presence or not of heparin (10 µg/ml). Calcein-labeled THP-1 cells (400,000 cells/insert) were placed in the upper chamber and allowed to migrate across the endothelial monolayer for 90 min towards the monocyte chemoattractant protein-1 (MCP-1, 50 ng/ml) added in the lower chamber. Control wells were filled with medium only. Results are expressed as transmigration index, calculated by dividing the average count of migrated cells towards the MCP-1 gradient by the average count of migrated cells in the control wells. Data are given as mean ± SEM

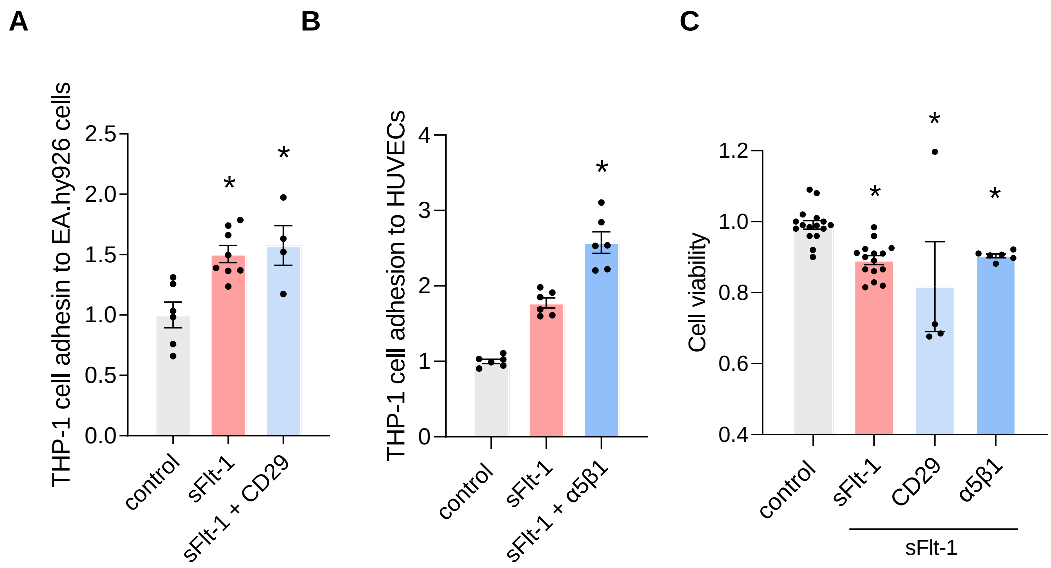

Figure S3. Endothelial cell adhesiveness to monocytes and viability under excess sFlt-1. EA.hy926 cells (**A**) or HUVECs (**B, C**) were treated with recombinant sFlt-1 (VEGFR1-Fc; 2 µg/ml) or control protein (IgG-Fc; 2 µg/ml) for 24h. As indicated, cells were also pre-treated (30 min) and co-incubated with the function-blocking antibodies to β1 and α5β1 integrins (CD29 clone Mab 13, 20 µg/ml; α5β1 clone JBS5, 10 µg/ml). (**A, B**) Monocyte adhesion to endothelial cells. (**C**) Cell viability determined using the MTT assay. The function-blocking antibodies didn't protect against sFlt-1 adverse effects. Results are expressed relative to control, and data are given as mean ± SEM. * $p < 0.05$ vs. control. Kruskal-Wallis and Dunn's multiple comparisons tests were applied
